# Understanding and preventing suicide in cancer patients from a therapeutic assessment and risk management perspective

**DOI:** 10.1192/j.eurpsy.2026.10657

**Published:** 2026-07-31

**Authors:** A. K. Ekwall

**Affiliations:** Health Sciences, Lund, Sweden

## Abstract

**Introduction:**

Suicide remains a major global public health concern, and cancer is associated with a significantly increased risk of suicide compared with the general population. The psychological, existential, and social consequences of a cancer diagnosis—including anxiety, depression, reduced quality of life, and feelings of burden—make suicidal ideation a critical yet often under-recognized issue. While patients with cancer frequently discuss death and dying, direct conversations about suicidal thoughts are uncommon.

**Objectives:**

To explore how the shift from suicide prediction to therapeutic assessment and risk management can be applied in the context of cancer care by using scientific literature from oncology as an application of the model in a theroetical paper.

**Methods:**

Drawing on Hawton et al.’s model of therapeutic assessment and risk management instead of prediction (2022)- a framework comprising predisposing, future, modifiable, and protective factors is presented and contextualized for cancer care by integrating evidence from oncology research. Scientific literature with studies from the oncology care was sought for each factor. Articles in English, covering different aspects of being a cancer patient, and not older than 10 years, were included.

**Results:**

Suicidal ideation represents a significant but often underacknowledged dimension of cancer care, affecting both patients and the healthcare professionals who provide support throughout the illness trajectory. It was shown that Hawton’s model is a useful tool för understanding the situation with life threatening diagnosis and suicidal thoughts. Incorporating systematic inquiry into suicidal ideation within routine cancer care encounters may normalize discussions of suicide, reduce stigma, and facilitate earlier identification of distress. The potential outcomes include a reduction in patient and professional suffering and a strengthened capacity of the healthcare system to prevent suicide.

**Conclusions:**

This framework provides clinicians with a way to explore not only the risks that may increase vulnerability but also the strengths and resources that can support resilience. Structured conversations about suicide extend beyond risk management. They affirm life by fostering hope, reducing stigma, and ensuring that patients with cancer feel acknowledged, supported, and understood throughout their illness journey.

Reference: Hawton, K., Lascelles, K., Pitman, A., Gilbert, S., & Silverman, M. (2022). Assessment of suicide risk in mental health practice: shifting from prediction to therapeutic assessment, formulation, and risk management. *Lancet Psychiatry*, *9*(11), 922–928. https://doi.org/10.1016/s2215-0366(22)00232-2

**Disclosure of Interest:**

None Declared

